# Device-Training for Individuals with Thoracic and Lumbar Spinal Cord Injury Using a Powered Exoskeleton for Technically Assisted Mobility: Achievements and User Satisfaction

**DOI:** 10.1155/2016/8459018

**Published:** 2016-08-17

**Authors:** Thomas Platz, Annett Gillner, Nicole Borgwaldt, Sylvia Kroll, Sybille Roschka

**Affiliations:** BDH-Klinik Greifswald, Neurorehabilitation Centre and Spinal Cord Injury (SCI) Unit, Ernst-Moritz-Arndt-Universität Greifswald, Karl-Liebknecht-Ring 26A, 17491 Greifswald, Germany

## Abstract

*Objective*. Results of a device-training for nonambulatory individuals with thoracic and lumbar spinal cord injury (SCI) using a powered exoskeleton for technically assisted mobility with regard to the achieved level of control of the system after training, user satisfaction, and effects on quality of life (QoL).* Methods*. Observational single centre study with a 4-week to 5-week intensive inpatient device-training using a powered exoskeleton (ReWalk*™*).* Results*. All 7 individuals with SCI who commenced the device-training completed the course of training and achieved basic competences to use the system, that is, the ability to stand up, sit down, keep balance while standing, and walk indoors, at least with a close contact guard. User satisfaction with the system and device-training was documented for several aspects. The quality of life evaluation (SF-12v2*™*) indicated that the use of the powered exoskeleton can have positive effects on the perception of individuals with SCI regarding what they can achieve physically. Few adverse events were observed: minor skin lesions and irritations were observed; no falls occurred.* Conclusions*. The device-training for individuals with thoracic and lumbar SCI was effective and safe. All trained individuals achieved technically assisted mobility with the exoskeleton while still needing a close contact guard.

## 1. Introduction

The wheelchair remains the primary option for mobility for persons with spinal cord injury (SCI) and lower-limb paresis.

So far, unpowered mechanical ortheses such as passive mechanical hip-knee-ankle-foot orthoses have been available to enable individuals with SCI to ambulate overground [1–6]. They can be regarded as training tools for selected individuals with SCI; they are, however, not suitable for routine mobility use because of the high energy cost required to ambulate, difficulty in standing and sitting, and the inability to climb stairs [1, 6]. Similarly, devices that use functional electrical stimulation (FES) alone or in combination with mechanical ortheses have been used for technology-assisted “walking” in individuals with SCI, again without becoming an option for everyday ambulation purposes [7–10].

A more recent technology for ambulation in individuals with SCI is powered exoskeletal devices that enable gait in individuals with SCI [11–18]. A major advantage over prior passive ortheses is that they are powered and can provide coordinated and controlled joint movements rather than rigid knee and ankle fixation.

A recent meta-analysis of the available published research on the clinical effectiveness and safety of powered exoskeletons used with individuals with SCI reported a total of 14 included studies (eight investigating the exoskeleton ReWalk, three Ekso*™*, two Indego®, and one unspecified exoskeleton) representing 111 patients [19]. Training programs were typically conducted three times per week (60–120 minutes per session) for 1–24 weeks. Following the exoskeleton training programs, 76% of individuals with SCI (95% confidence interval [CI]: 59%–90%) were able to ambulate with no physical assistance. In five studies, 38% (95% CI: 19%–59%) of patients reported decreases in spasticity with exoskeleton training; in three studies, 61% (95% CI: 20%–95%) of patients reported improvements in bowel movement regularity with exoskeleton training. No serious adverse events occurred. The incidence of fall at any time during training was 4.4% (95% CI: 1.0%–10.0%), all occurring while tethered using a first-generation exoskeleton and none resulting in injury. The incidence of bone fracture during training was 3.4%. The authors concluded that powered exoskeletons allow individuals with SCI to ambulate safely in real-world settings.

Yet, aside from the assuring potential of these devices to enable technology-assisted walking in individuals with SCI [11–19], there is still a lack of knowledge regarding their clinical use.

Who are the individuals with SCI that might be able to use a powered exoskeleton and how can they be selected? What are the results of a structured device-training, that is, the “milestones” achieved such as standing up and sitting down, walking a few meters, or walking moderate distances with an exoskeleton, and how quickly can they be achieved? How satisfied are users with the device-training and the use of a powered exoskeleton after training? Are there adverse events such as falls and skin lesions or pain associated with the use of the device? Are there any effects on quality of life when using a powered exoskeleton?

This observational study set forth a clinical pathway for user selection and inpatient device-training with an exoskeleton. Results of the device-training in terms of the achieved milestones for device use, user satisfaction, and effects on quality of life were documented.

## 2. Materials and Methods

### 2.1. Study Design

A prospective, single-group observational study was conducted at the SCI Centre of the BDH-Klinik Greifswald. The study specifications were in accordance with the Declaration of Helsinki (1964) and were based on the human subjects' understanding and consent after the local Ethical Committee had approved the study.

### 2.2. Participants

#### 2.2.1. Inclusion Criteria

Inclusion criteria are as follows:

1. Males or females with American Spinal Injury Association (ASIA) Impairment Scale (AIS) A, B, or C paraplegia [21] resulting from SCI (traumatic or disease-related) at thoracic or lumbar level without the ability to actively stand or walk (with or without devices).
2. Duration of SCI ≥ 3 months.
3. Spinal column considered stable without specified movement restriction.
4. Ages from 18 to 75 y.
5. Height from 170 to 190 cm.
6. Weight <100 kg.
7. Ability to give informed consent.

#### 2.2.2. Exclusion Criteria

Exclusion criteria are as follows:

1. Diagnosis of neurological injury other than SCI including
  - multiple sclerosis (MS),
  - stroke,
  - cerebral palsy (CP),
  - amyotrophic lateral sclerosis (ALS),
  - traumatic brain injury (TBI),
  - Parkinson's disease (PD),
  - other neurological conditions that the study physician considers in his/her clinical judgment to be exclusionary.
2. Severe concurrent medical disease, illness, or condition.
3. Lower extremity fracture within the past 6 months.
4. Trunk and/or lower extremity pressure ulcers.
5. Severe spasticity (defined by an Ashworth score of 4 for hip, knee, and/or foot flexors and/or extensors at rest or clinical impression of the study physician or physiotherapist, especially in the case of Ashworth score of 3 for hip, knee, and/or foot flexors and/or extensors at rest).
6. Significant contractures defined as flexion contracture limited to ≥ 35° at the hip and ≥ 20° at the knee.
7. Diagnosis of heterotrophic ossification of the lower extremities.
8. Psychopathology documentation in the medical record or history of that which may conflict with study objectives.
9. Other illnesses that the study physician considers in his/her clinical judgment to be exclusionary.
10. Pregnancy and/or lactating females.

#### 2.2.3. Financial Coverage for the Inpatient Device-Training

Eligible individuals with SCI needed to have financial coverage for the inpatient device-training. Financial coverage for inpatient training for individuals with SCI depended on individual health and social security circumstances. Once individuals with SCI were assessed as eligible for an exoskeleton device-training, financial coverage for their inpatient device-training had to be clarified on an individual basis before training could commence.

### 2.3. Patient Selection and Clinical Pathway

The clinical pathway for patient selection and the exoskeleton training had the following steps:

1. Information leaflet and questionnaire.
2. Inpatient interprofessional assessment.
3. Inpatient training.
4. Evaluation.

#### 2.3.1. Information Leaflet and Questionnaire

The SCI centre in Greifswald was the first in Germany to offer an exoskeleton device-training for technically assisted mobility. Individuals with SCI who learnt about this new technology by public media and developed an interest in using the device contacted the SCI centre. They were sent written information about the exoskeleton and device-training together with a questionnaire asking for medical information. Once they had returned the questionnaire, they were again informed whether the exoskeleton might individually be an option for them. In case they were potential candidates, they were invited for an overnight interprofessional assessment.

#### 2.3.2. Inpatient Interprofessional Assessment

Within the SCI centre, an “exoskeleton team” had been built which assessed (and later trained) exoskeleton user candidates during 24 hrs. Inpatient visit included a physician (TP), two physiotherapists (AG and NB), a sport therapist, and a psychologist (SK).

Physiotherapists and sport therapist explained the device to the candidates, its options, and the training and checked prerequisites for a training such as leg length, passive joint mobility, spasticity, skin condition, and alike. The psychologist evaluated the candidate's motivation to use the device, the degree of psychological stability, and any distress from having SCI, as well as the implications of the intended exoskeleton use for the candidate's psychosocial situation. The physician assessed aspects of physical and mental health related to the SCI and the exoskeleton use and again explained the device, the training, the evaluation of it, and future steps. After consultation and exchange within the team, the physician informed the candidate about the results and made a shared decision with the candidate regarding the exoskeleton use. When a positive decision was made, the inpatient training was planned.

#### 2.3.3. Inpatient Training

The inpatient training was planned for a period of 4-5 weeks. It included a daily exoskeleton training (60 minutes, Monday through Friday) and further therapeutic exercises as individually indicated.

The skills to be learned during the exoskeleton training included (1) sit-to-stand, (2) stand-to-sit, (3) 2-arm standing balance, (4) 1-arm standing balance, (5) walking straight ahead, (6) walking in a curve, when stable indoor overground walking capability was gained, (7) stair climbing (individual cases), and (8) outdoor walking.

For the exoskeleton training, the institutional type of the ReWalk powered exoskeleton was used [12–18]. All subjects were individually fitted to the exoskeleton according to pelvic width, thigh length, and shank length. The pelvic band size was determined according to the width of the user's waist. Participants wore their own shoes with the device.

Once the participant was fitted properly in the device, he or she participated in learning the maneuvers of standing up, sitting down, standing balance, and weight shifting as prerequisites for walking in the device. Once standing, walking is accomplished with a combination of body position, dynamic trunk posture, weight shifting, and arm/crutch placement. In addition, the system has a mode to support ascending and descending stairs.

Two therapists were continuously present to provide hands-on and/or verbal assistance as individually needed. The amount of assistance provided was determined as either “close contact guard” (CCG) (physical help needed), “minimal assist” (MA) (mainly verbal assistance, occasional physical help), or “no assist” (NA).

#### 2.3.4. Evaluation

The achievement of milestones (see below: Assessment, Milestones) was continuously monitored. The satisfaction with the training and the use of the device were assessed at the end of training. Before the training commenced, after the training, and 4 weeks later self-perceived quality of life was documented. A semistructured interview with the psychologist (SK) helped the user to reflect the achievements of the training. A final talk with the physician (TP) rounded the evaluation up and led to a shared decision regarding future activities (ambulatory training, perspective of provision with a personal device).

### 2.4. Assessment/Outcome Measures

#### 2.4.1. Device-Training Milestone Achievements

Documented was the number of device-training sessions until the following milestone had been achieved:

- Sit-to-stand.
- Stand-to-sit.
- Standing balance for 1 minute with both crutches.
- Walk 10 meters straight.
- Walk 10 meters straight and in curve.
- Ascend, turn around, and descend a flight of 12 stairs.
- Walk 500 meters (outdoors).

For each aspect it was documented when it was achieved with either a “close contact guard” (CCG) (physical help needed), “minimal assist” (MA) (mainly verbal assistance, occasional physical help), or “no assist” (NA).

#### 2.4.2. Satisfaction Questionnaire

A user's satisfaction questionnaire with ten items was used [18]. The participants were asked to respond to 10 statements concerning the use of the device. They provided their subjective opinion by indicating in a Likert scale the number that best represented how they felt: (1) strongly disagree, (2) disagree, (3) somewhat agree, (4) agree, and (5) strongly agree. All of the statements were phrased in a positive manner regarding the training process, comfort, safety of use, and medical issues (such as pain, spasticity, bowel movements, and breathing).

The items were as follows.

*Sat 1*. Training/learning to use the device is not complicated.

*Sat 2*. Wearing/adjusting the device is relatively simple.

*Sat 3*. It was comfortable to exercise with the device.

*Sat 4*. The usage of the device did not cause considerable pain.

*Sat 5*. I did not feel excessive fatigue while exercising with the device.

*Sat 6*. After completing the training period I felt comfortable using the device.

*Sat 7*. Training with the device diminished spasticity in my legs.

*Sat 8*. I did not have breathing difficulties while training with the device.

*Sat 9*. I felt improvement in my bowel movement during the training program.

*Sat 10*. After completing the training I felt safe using the device.

#### 2.4.3. Quality of Life (SF-12v2 Health Survey)

The SF-12v2*™* Health Survey (Version 2.0) acute (1-week) recall version for self-administration [22, 23] was used to assess various aspects of quality of life (QoL). The acute form of the SF-12 was designed for applications in which health status would be measured weekly or biweekly. In this study, it was applied before and after the course of inpatient device-training and 4 weeks later (follow-up).

The SF-12v2 form with its 12 items provides an eight-domain profile of scales, that is, physical functioning (PF), role physical (RP), bodily pain (BP), general health (GH), vitality (VT), social functioning (SF), role emotional (RE), and mental health (MH), in addition to the summary measures, physical and mental component summary scales (PCS-12 and MCS-12). Normalization algorithms had been developed for all eight scales using data from the 1998 general US population [23] and were used for the study.

#### 2.4.4. Further Outcome Measures

Additional outcome measures were adverse events, that is, falls, cardiovascular events, skin lesions, joint problems, and pain.

There were some changes in motor or sensory scores (International Standards for Neurological Classification of Spinal Cord Injury, ISNCSCI) [21], spasticity scores (Resistance to Passive Movement Scale, REPAS) [24], and passive motility of leg joints from before to after testing after training. REPAS is a validated spasticity scale that is based on the Ashworth scale and provides summary scores for spasticity/resistance to passive movements across joints, that is, for limbs.

### 2.5. Statistical Analyses

Descriptive statistics were used.

Baseline characteristics are given as number or mean (SD) [minimum and maximum] as appropriate.

Outcome measures (milestones, user satisfaction, and quality of life domains) are presented as mean and 95% confidence intervals (CI). Thereby, statistical inference is facilitated for the evaluation: whether group mean scores deviate from a predetermined level of interest for both the satisfaction questionnaire and the quality of life measures.

#### 2.5.1. User Satisfaction Questionnaire

For the 5-step ordinal scale with the possible responses of (1) strongly disagree, (2) disagree, (3) somewhat agree, (4) agree, and (5) strongly agree, it was of interest to note whether satisfaction (scores >3) or dissatisfaction (scores <3) was on average signaled by the exoskeleton users for the aspects addressed. If both mean and 95% CI were above or below 3, statistically significant satisfaction or dissatisfaction could be assumed for the group; for example, a mean [95% CI] of 4.2 [3.7–4.7] would denote satisfaction among the exoskeleton users, while a mean [95% CI] of 2.3 [1.8–2.8] would denote dissatisfaction, and a mean [95% CI] of 2.7 [2.3–3.1] would indicate neither of the two.

#### 2.5.2. Quality of Life (SF-12v2)

(Raw) scores of the SF-12v2 [22] had been recoded to indicate better QoL with higher scores; the item general health had been recalibrated to support a linear relationship between item scores and the underlying health concept; scale items had then been aggregated for the 8 domains; these scores had been *z* score standardized based on 1998 general US population data; and, finally, the aggregating scales for physical and mental health had been calculated [23].

Deviations of these *z* scores among the exoskeleton users from 0 denote either a statistically significantly higher (mean and 95% CI > 0) or lower (mean and 95% CI < 0) QoL in the respective domain when compared to the norm applied.

Change scores for SF-12v2 *z* standardized domain scores were calculated as postscores − prescores and 4-week follow-up − prescores, respectively. Deviations of these change scores among the exoskeleton users from 0 denote either a statistically significantly improvement (mean and 95% CI > 0) or deterioration (mean and 95% CI < 0) of their QoL in the respective domain.

## 3. Results

### 3.1. Exoskeleton Trainee Selection Procedure

**Figure 1:**
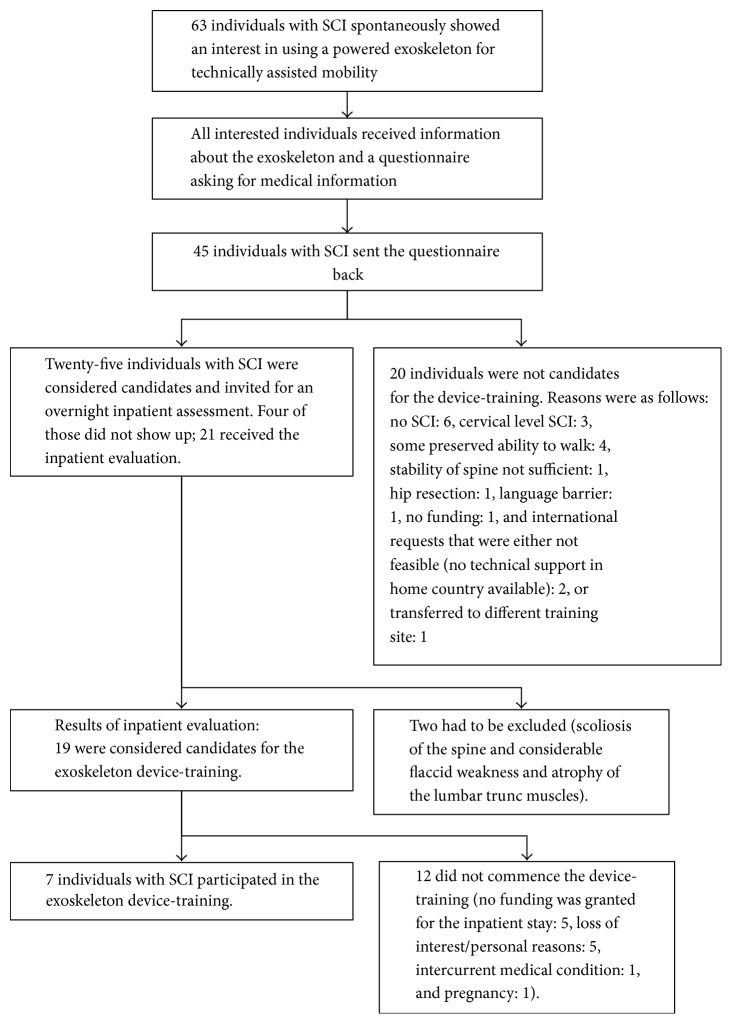
Exoskeleton trainee selection.

Secondary to mass media attention to the topic of exoskeleton technology for technically assisted “walking” in nonambulatory individuals with SCI, 63 individuals with SCI contacted the SCI centre in Greifswald with an interest in using such a powered exoskeleton. A two-step screening approach, that is, questionnaire-based and inpatient evaluation, identified 19 candidates for the device-training, of whom 7 took part in the training (for details of the selection procedure, see Figure 1).

### 3.2. Patient Characteristics

Among the participants (compare Table 1), there were more male than female individuals with SCI; they had mainly a complete SCI (AIS A), with the highest SCI level being T5 and the lowest L1. Spasticity was only mild in the trained group.

### 3.3. Achievement of Training Milestones

During the 4-week to 5-week course of daily training (Monday to Friday), all participants learnt to stand up and sit down, keep balance while standing, and walk indoors (“10 m straight and in curve”) with the exoskeleton, at least when physical help was granted (compare Table 2). Climbing a flight of stairs with a close contact guard (CCG) was, however, only achieved by 4 out of 7 individuals.

Within two training sessions, sit-to-stand and the reverse as well as balance while standing with the exoskeleton were achieved when the help of a CCG was provided. Walking indoors with a CCG took about 2 weeks.

Carrying out these activities without the need for continued physical help, that is, with mainly verbal assistance and occasional physical help (minimal assist, MA), was not achieved by all but was achieved by the majority of participants for sit-to-stand and the reverse as well as balance activities within 2 to three weeks of daily training. Walking with MA was only achieved by the minority of trainees within the training course.

Most trained individuals (5 of 7) achieved “standing balance for 1 minute with both crutches” without need for any assistance (no assist, NA), while the other activities with the exoskeleton still needed some assistance in almost all participants at the end of the training course.

### 3.4. User Satisfaction with the Exoskeleton Device-Training

**Figure 2:**
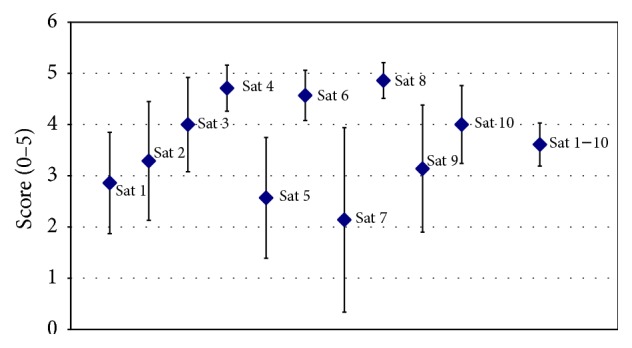
Satisfaction with the exoskeleton device-training. Presented are group (*N* = 7) mean values (rhomb) and 95 confidence intervals (whiskers) for each of the 10 items of the training satisfaction questionnaire (Sat 1 to Sat 10) and the individual average across all 10 items (Sat 1–10). For the questions addressed (Sat 1 to Sat 10), see text in Section 2.

For half of the items (i.e., 1, 2, 5, 7, and 9) the confidence intervals included the middle score of 3 indicating neither a high degree of satisfaction nor dissatisfaction with the device-training (compare Figure 2). Based on the descriptive statistics applied (mean and 95% CI < 3), no item signaled on average dissatisfaction.

Above average satisfaction (mean and 95% CI > 3) had been observed for items 3, 4, 6, 8, and 10 and the summary score for all items. Thus, overall, there was a fair degree of satisfaction with the training (Sat 1–10). On average, the participants judged that it was comfortable to exercise with the device (Sat 3) and they felt comfortable to use it after the training period (Sat 6); they further indicated that its use did not cause considerable pain (Sat 4) or breathing difficulties (Sat 8), and they felt safe using the device after completing the training (Sat 10).

### 3.5. Quality of Life (SF-12v2)

**Figure 3:**
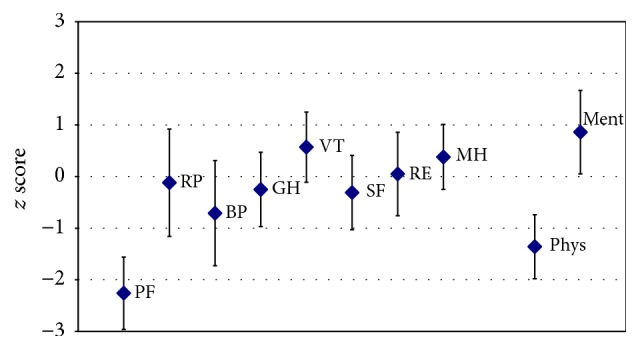
Quality of life at the commencement of the exoskeleton device-training. Presented are baseline group (*N* = 7) mean values (rhomb) and 95 confidence intervals (CI) (whiskers) for each of the 8 domains of the SF-12v2, that is, physical functioning (PF), role physical (RP), bodily pain (BP), general health (GH), vitality (VT), social functioning (SF), role emotional (RE), and mental health (MH), in addition to the summary measures, physical and mental component summary scales (Phys and Ment). The SF-12v2 data presented had been *z* score standardized based on 1998 general US population data. When both mean and CI are <0, a lower than average QoL in that domain is documented, while there is higher than average QoL when both mean and CI are >0. When the CI includes 0, the group did not significantly deviate from the norm. While, for most domains, the groups' data did not significantly deviate from the norm, physical functioning (PF) and the physical component summary scale were significantly lower in the group compared to the norm. Conversely, the mental component summary scale indicated a higher QoL among the trained individuals with SCI.

When assessed at baseline (before the device-training commenced), for most domains, the groups' QoL data did not significantly deviate from the norm (compare Figure 3). Physical functioning (PF) and the physical component summary scale (Phys) were, however, significantly lower in the group compared to the norm. Conversely, the mental component summary scale (Ment) indicated a higher QoL among the trained individuals with SCI in that respect.

Changes scores were calculated as after device-training minus before device-training scores and indicated changes of QoL over the 4 to 5 weeks of exoskeleton device-training.

**Figure 4:**
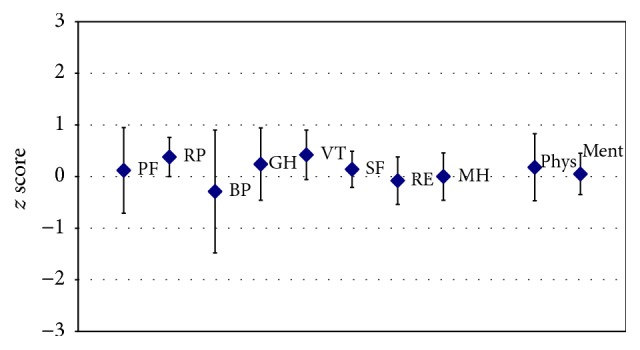
Quality of life: changes from before to after exoskeleton device-training. Presented are group (*N* = 7) mean change scores (posttest − pretest) (rhomb) and 95 confidence intervals (CI) (whiskers) for each of the 8 domains of the SF-12v2 (for explanation of abbreviations, see legend of Figure 3). The SF-12v2 change scores presented had been *z* score standardized. When both mean and CI are <0, a significant deterioration of QoL in that domain is documented, and there is a significant improvement of QoL when both mean and CI are >0. When the CI includes 0, the groups' QoL did not significantly change after training. Note, for role physical (RP), a significant improvement from before to after test was documented. None of the other domains or summary measures indicated a significant change.

For the SF-12v2 domain role physical (RP), a significant improvement from pretest to posttest was documented (*z* score mean [95% CI]: 0.38 [0.01–0.76]) (compare Figure 4). None of the other domains or summary measures indicated a significant change.

Changes scores were calculated as 4 weeks after device-training minus before device-training scores and indicated changes of QoL over the 4 to 5 weeks of device-training and a consecutive 4-week follow-up period at home compared to baseline data before exoskeleton device-training commenced.

**Figure 5:**
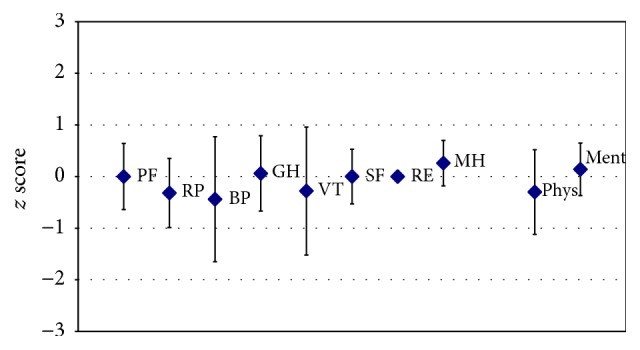
Quality of life: changes from before device-training to follow-up 4 weeks after exoskeleton device-training. Presented are group (*N* = 7) mean change scores (follow-up 4 weeks after training − pretest) (rhomb) and 95 confidence intervals (CI) (whiskers) for each of the 8 domains of the SF-12v2 (for explanation of abbreviations, see legend of Figure 3). The SF-12v2 change scores presented had been *z* score standardized. When both mean and CI are <0, a significant deterioration of QoL in that domain is documented, and there is a significant improvement of QoL when both mean and CI are >0. When the CI includes 0, the groups' QoL did not change significantly after training. Note, for none of the domains or summary measures, a long-term change in QoL was documented.

None of the SF-12v2 domains or summary measures indicated a significant change over this extended period (compare Figure 5).

### 3.6. Adverse Events

Adverse events were infrequent and mild. No falls were observed during the device-training. No unwanted cardiovascular effects were documented. Skin lesions were observed in 4 subjects. They consisted of short-term mild pressure or friction symptoms around or below the knee level, which needed adjustment of the device fixation and/or padding; discontinuation of the training was not necessary. Mild pain during the training, for example, of the shoulder girdle, trunk, and/or arm muscles, was reported in 2 subjects. Swelling was observed in 2 subjects (activated osteoarthritis of the knee in one patient, some redness and a mild swelling over the lumbar spine in another patient thought to be caused by local mechanical irritation); these conditions could be managed by medical treatment and did not imply discontinuation of the training.

### 3.7. Other Effects

Group data did not indicate a systematic change of the SCI motor or sensory signs (ISNCSCI), spasticity (REPAS), or activities of daily living competence (SCIM).

In two subjects, neuropathic pain in the legs diminished during the training. In one of these subjects, neuropathic pain was severe enough (despite of medication) that the subject had had a disturbed night sleep each night for many years. Over the course of the exoskeleton device-training, the pain was almost completely gone with great relief for his nights.

## 4. Discussion

This observational study used a clinical pathway for user selection and inpatient device-training with an exoskeleton for nonambulatory individuals with thoracic and lumbar spine SCI. Results of the device-training in terms of the achieved milestones for technically assisted mobility, user satisfaction, and effects on quality of life were documented.

The structured clinical pathway included a two-step selection procedure and an inpatient device-training.

The first step with written information about the exoskeleton and a questionnaire based on selection criteria for the exoskeleton use identified 25 potential candidates for the training out of 63 individuals who had spontaneously contacted the SCI centre. They were then invited for the second selection step, the interprofessional inpatient evaluation (compare Figure 1). Thereby, 19 candidates for the training could be confirmed. Seven of these candidates participated in the device-training; among all individuals with SCI who had shown an interest, the percentage was 12%. Overall, reasons for exclusion that were related to the SCI (no SCI, cervical SCI, weakness and atrophy of the lumbar trunk muscles, and at least some preserved ability to walk) were documented in 14 out of 63 individuals who contacted the SCI centre, that is, 22%. Lack of funding was a reason not to participate in the device-training in 6 out of 63 individuals, that is, 10%.

All seven subjects who were identified as candidates and started the training completed the inpatient device-training as scheduled.

### 4.1. Achievements during the Course of Device-Training

A daily (60 minutes) exoskeleton training for 4 to 5 weeks was sufficient for all participating individuals with SCI to learn to stand up and sit down, keep balance while standing, and walk indoors (“10 m straight and in curve”) with the exoskeleton, at least when physical help was granted.

Thus, a major step in competence to use the system was achieved by the type, structure, and schedule of training provided.

Indeed, while in the beginning the presence and help of 2 therapists had been provided and was necessary, the first successful achievements were quickly observed: within two training sessions, sit-to-stand and the reverse as well as balance while standing were achieved with the help of a close contact person (CCG). Walking indoors with a CCG took about 2 weeks. At that time physical help was only necessary by one therapist.

While these achievements were considerable and were reached by all trained individuals, an ongoing more prolonged training seemed necessary to learn to use the system proficiently. Thus, it is not unexpected that limitations of achievements after the course of training were also evident: climbing a flight of stairs with a close contact guard (CCG) was only achieved by 4 out of 7 participants. Further, carrying out the activities of standing up and sitting down, keeping balance while standing, and walking indoors without the need for continued physical help, for example, mainly verbal assistance and occasional physical help (minimal assist, MA), had not been achieved by all trained individuals. Still, the majority of participants achieved sit-to-stand and the reverse as well as balance activities under MA within 2 to three weeks of daily training and standing balance even without need for any assistance (no assist, NA). Walking with MA or even NA was, however, only achieved by single trainees within the time span of training provided.

### 4.2. User Satisfaction with the Device-Training and Exoskeleton Use

User satisfaction with the device-training had been measured with a questionnaire that had been collated for that purpose [18]. While this 10-item collection had not formally been evaluated, it has some face validity for its use with exoskeleton use and training.

On average, the participants felt comfortable to exercise with the device and to use it after the training period; importantly, they felt safe using the device after completing the training. In addition, they indicated that its use did not cause considerable pain or breathing difficulties. According to the participants' perception spasticity and bowel movements were, however, on average not improved. The latter results are not congruent with previous reports [19]; with regard to spasticity, they can be explained by the low level of spasticity in the trained individuals (compare Table 1).

Overall, the grand average of the user satisfaction questionnaire (average across all questions, compare “Sat 1–10” in Figure 2) was positive indicating a fair user satisfaction. Noteworthy, this grand average is not based on a formal test construction but can still provide a central tendency of the questionnaire results.

### 4.3. Quality of Life

The SF-12v2 form with its 12 items was used to assess QoL in eight domains, that is, physical functioning (PF), role physical (RP), bodily pain (BP), general health (GH), vitality (VT), social functioning (SF), role emotional (RE), and mental health (MH), in addition to the summary measures, physical and mental component summary scales (PCS-12/Phys and MCS-12/Ment).

When assessed at baseline (before the device-training commenced) the groups' data did not significantly deviate from the norm for most domains (compare Figure 3). Physical functioning (PF) and the physical component summary scale (Phys) were, however, significantly lower in the group compared to the norm. Conversely, the mental component summary scale (Ment) indicated a higher QoL among the trained individuals in that respect.

Thus, these individuals with SCI judged their own physical functioning lower than the general population, an effect that is not unexpected given their paraparesis. At the same time, the mental component summary scale indicated a higher psychoemotional stability and wellness than observed in the general population. A likely explanation is that individuals with SCI who show an interest in the use of an exoskeleton to “overcome” their only wheelchair-bound mobility are a group of individuals with a high degree of psychoemotional stability and resources. This competence again is regarded as one determinant of a successful training and exoskeleton use in the long run. It is highly recommended to address motivational, psychosocial, and emotional aspects when counselling individuals with SCI with a desire to use an exoskeleton for technically assisted walking.

It was further of interest whether the training itself changed the self-perceived QoL either immediately after the training or after some time (4 weeks) when being back in the individual home situation. Most SF12 domains did not indicate (statistically significant) systematic changes in the group of trained individuals with SCI, with one exception: physical role (how much one thinks she or he can accomplish and how much one feels to be limited in the kind of work or other activities) was on average improved (compare Figure 4, RP) (*z* score mean [95% CI]: 0.38 [0.01–0.76]). Thus, the self-perception of possible achievements versus limitations with the conduct of activities was positively changed after the device-training. There was, however, no long-term effect of the training on QoL once it was discontinued (compare Figure 5).

It is well conceivable that standing and being mobile in an upright posture and to experience the possibility to “overcome” a purely wheelchair-based mobility gave the trained individuals with SCI a different perspective on what they can physically achieve.

Another body function that was reported as improved in two participants was neuropathic pain in the affected legs.

### 4.4. Adverse Events

Only minor adverse events were observed during the device-training. No fall occurred. Cardiovascular or respiratory problems were not observed in the trained group. Skin irritations were, however, observed. Since individuals with SCI are prone to pressure ulcers, it cannot enough be stressed that the occurrence of skin irritations and lesions needed close attention during the device-training. Here, they could be managed by changing the individual device fixation (bands) or padding. Some pain/discomfort of shoulder girdle, trunk, and arm muscles was occasionally observed, especially when training commenced and users were uncertain how to perform the activities with the exoskeleton and exerted more power than necessary or used their musculature in a different way than usually.

### 4.5. Study Limitations

This observational study was based on a small number of participants. Accordingly, the data is not necessarily representative for other individuals with SCI.

For reasons of comparison across the various aspects observed and the documented changes over time parametric descriptive statistics were used, that is, mean and 95% confidence intervals. For the user satisfaction questionnaire with its ordinal scale items [18] equal distances between response levels around the centre of 3 were assumed.

For the SF-12v2 data, the suggested normalization algorithm was applied; the algorithm used data from the 1998 general US population [23], while here German participants had been enrolled.

### 4.6. Comparison with Other Clinical Evaluations of Exoskeleton-Assisted Walking in Individuals with SCI

Study populations in other clinical trials were similar with relatively small numbers of subjects (median 7), a preponderance of male subjects (83%), and mostly complete SCI [19]. Training volume varied considerably across clinical trials in terms of number of training sessions, session length, and overall program duration. The reported inpatient device-training was shorter (4 to 5, instead of 6 to 24 weeks) yet more intense (5 sessions per week compared to 1 to 3 sessions per week) than most other reported training schedules (e.g., [11–13, 15, 16]). All trials used indoor walking [11–18]; only few (e.g., [12]) included outdoor walking and stairs during the device-training as in this report. Overall, approximately 3/4 of trainees achieved technically assisted ambulation with an exoskeleton in the clinical trials reported; as in this study, adverse events had been reported to be mild [19]. User satisfaction in this report showed a comparable pattern as previously documented [18].

While the results of this study cannot be directly compared to other device-training schedules, the data favours the notion that this short-term intensive inpatient device-training had by and large comparable clinical effectiveness. The refined documentation of achieved milestones and QoL aspects adds to our knowledge about the effects of an exoskeleton device-training in nonambulatory individuals with SCI.

## 5. Conclusions

Provision of 4-week to 5-week intensive daily inpatient exoskeleton device-training provided by two experienced physiotherapists and sport therapist was successful in each trained individual in the sense that standing up and sitting down, keeping balance while standing, and walking indoors activities could be performed with the exoskeleton system at least when close physical contact by therapists was provided. For some activities and subjects, minimal or even no assist was sufficient after training. Climbing stairs and walking outdoors had rather been the exception during that time span.

Assessing user satisfaction with a questionnaire indicated that the participants felt comfortable using the exoskeleton during the training and safe after the training.

A formal QoL evaluation indicated that the training and actual use of the system can have positive effects on what individuals with SCI perceive they can achieve physically.

Adverse events were minor and were related to skin irritations and musculoskeletal symptoms during training.

## Figures and Tables

**Table 1:** Patient characteristics (*N* = 7).

Sex, female/male, *N*	2/5
Age, *mean (SD) [min.–max.]*	48.3 (10.2) [33–58]
Duration of SCI in years, *mean (SD) [min.–max.]*	11.4 (10.1) [2–29]
ASIA Impairment Scale (AIS) (*N*)	A (6), C (1)
ISNCSCI single neurological level (*N*)	T5 (2), T10 (2), T11 (1), T12 (1), L1 (1)
ISNCSCI motor, upper limb (max. 50), *mean (SD)*	50 (0)
ISNCSCI motor, lower limb (max. 50), *mean (SD)*	3 (4)
ISNCSCI, sensory (max. 112), *mean (SD)*	68 (15)
REPAS leg (max. 40), *mean (SD)*	4 (8)
SCIM (max. 100), *mean (SD)*	72 (3)
Height, *mean (SD) [min.–max.]*	176 (7) [168–188]
Body weight, *mean (SD) [min.–max.]*	72.6 (11.4) [56–88]

ASIA, American Spinal Injury Association; AIS, American Spinal Injury Association Impairment Scale; ISNCSCI, International Standards for Neurological Classification of Spinal Cord Injury; REPAS, Resistance to Passive Movement Scale; SCIM, Spinal Cord Independence Measure [20].

**Table 2:** Achievement of milestones during the exoskeleton training.

Number of training units until a milestone was individually achieved (presented as mean [95% confidence interval], number of subjects)		
Close contact guard (CCG) (physical help needed)	Minimal assist (MA) (mainly verbal assistance, occasional physical help)	No assist (NA)
Sit-to-stand		
1.3 [0.8–1.7] (*N* = 7)	10.2 [2.8–17.6] (*N* = 5)	12 (*N* = 1)
Stand-to-sit		
1.3 [0.8–1.7] (*N* = 7)	10.7 [5.1–16.2] (*N* = 6)	16 [12 & 20] (*N* = 2)
Standing balance for 1 minute with both crutches		
1.3 [0.8–1.7] (*N* = 7)	4.7 [2.9–6.5] (*N* = 7)	9.0 [5.2–12.8] (*N* = 5)
Walk 10 meters straight		
4.9 [2.1–7.7] (*N* = 7)	23 [21 & 25] (*N* = 2)	22 (*N* = 1)
Walk 10 meters straight and in curve		
8.1 [2.1–14.2] (*N* = 7)	21 (*N* = 1)	22 (*N* = 1)
Ascend, turn around, and descend a flight of 12 stairs		
18.5 [7.3–29.7] (*N* = 4)		
Walk 500 meters		
11 [7 and 15] (*N* = 2)	22 (*N* = 1)	
